# Scotch Physicians and Surgeons

**Published:** 1888-01-07

**Authors:** 


*(To be continued.)*

### SCOTCH PHYSICIANS AND SURGEONS.

### SIR JAMES Y. SIMPSON.

THE chief industry of Edinburgh is education. It would perhaps be rash to say that Edinburgh turns out scholars, talking in the same strain as that we employ in alluding to the industries of Manchester and Sheffield; because wise men, or even learned ones, cannot be made to order like scizzors and calico; and the action of the influences of culture on the raw material of human nature cannot be so safely predicted as the developments which the processes of manufacture bring out of cotton or steel. But it is undeniable that the best energy of Edinburgh is devoted to the intellectual development of youth, and that the great interests of the city are those of its educational institutions. At the head of these, as the most renowned and the most direct and specific in its action, stands the great Medical School of the University. Its fame is of no recent growth, and the yearly increasing number of students who seek admission to its portals shows that Edinburgh still keeps its old place among the great medical schools of the world. Such a reputation as it possesses cannot be gained, nor can it be maintained, without teachers of undoubted eminence—men who are not only themselves interested in their work, and determined to elucidate those scientific problems which beset all who travel the upward path of science, but who have also the art to imbue their students with a sympathetic enthusiasm. A good teacher of any branch of medical study must be not only an able practitioner and a thoughtful scientist himself; he must be able also to make those who learn from him able practitioners and thoughtful scientists too. Such men Edinburgh has never lacked, but of all the distinguished names that mark the roll of Edinburgh professors in the medical faculty, the most distinguished, the best known to the world at large, as well as to the profession, is that of James Simpson, whose fame as an obstetric physician had spread over the kingdom before the introduction by him of chloroform into surgical practice made him world-renowned.

James Young Simpson was born at Bathgate, about eighteen miles from Edinburgh, on June 7th, 1811. His father was a baker by trade, and the son of a small farmer; and though there was a far-off connexion on his mother's side with some blue-blooded family, he was distinctly a son of the soil, a man of the people. He was the eighth and youngest child of his parents, and was from his infancy regarded as the Benjamin of the flock. The care and affection of the whole family were lavished on him, and it was the united efforts of all that gave him the means to pursue that University education which was his first step up the ladder of fame. This sympathetic union of all members of a household to secure the good of one is in many respects characteristically Scotch. Elsewhere a man may push himself from a low position to a high one, but there will not often be seen all his brothers and sisters, as well as his parents, working and saving to provide him with the means of making his way. This would not be possible if the family affection which helps the aspirant at the beginning of his career were not returned by him to the end. But any neglect of, or ingratitude to, the humble kinsmen is exceedingly rare, and reflects injuriously on the reputation of him who is guilty of it; on the contrary, the successful brother is almost certain to help his kindred and their children to attain a position equal to his own. Thus Alexander Simpson, his father's successor in the baking business, was to the end his brother James's friend, adviser, confidant, and helper. When Alexander was ill, nothing would satisfy the busy Professor but that "Sandy" should come to Edinburgh and be nursed in his own house. When the crowning honour of a baronetcy was offered to him, Dr. Simpson wrote to his son: "Nobody will be prouder than your uncle at Bathgate;" and during his last illness, even with wife and children about him, it was on the elder brother's knee that Simpson's head lay, while he moaned at intervals, "Oh, Sandy, Sandy!" as if he were still the little brother asking for help from his senior in some childish ailment. In the early days of his professional career, when Simpson's fame was greater than his fortune, Sandy, though married and a father himself, was as ready as ever to help James with his hard-earned savings; and the letters which both he and his sister Mary, James's second mother, wrote to every one who in any way befriended their brother, show a deep sense of personal gratitude that prove how sincerely they felt that his interests were identical with their own.

It is necessary to insist on this point, not merely to show the proverbial "clannishness" of the Scotch nature, but to explain how it was possible for James Simpson to obtain the education necessary to qualify him for the position he ultimately attained. The son of a man who was long unfortunate in business, though from the date of James's birth things began to fall out more prosperously, exceptional advantages could not be secured for one member of the family without exceptional sacrifices on the part of all. It is true that the boy early showed remarkable intelligence—the power of concentration which enabled him to pursue his studies in an apartment which was at once shop and kitchen, was in itself an enviable gift; but the consciousness that his family believed in him, and expected great things of him, must have been to a man of James Simpson's nature a great inducement to energy and perseverance.

It says something for the boy's character that he could be trusted, at the age of fourteen, to enjoy the lonely independence of college life. It is true that Bathgate is only eighteen miles from Edinburgh; but that was a long distance in those days. How rustic the folks were within twenty miles of the capital where David Hume had erected a kingdom of elegant scepticism may be shown by two evidences of superstitions which Simpson remembered having seen in his youth. Once, when a murrain visited a prosperous dairy farm, a hapless cow was buried alive as a sacrifice to the spirit of the plague—with what result upon the pestilence is not recorded. Again, when an uncle of Simpson's took a farm, he walled round a small triangular patch of ground for the "Gudeman's Croft." The "Gudeman" was, of course, the Prince of Darkness, who was thus courteously entreated in order to induce him to spare the rest of the farm from blight, barrenness, and other evils that he was supposed to have dominion over. It is narrated, however, that the farmer, being a hard man, selected the most worthless and sterile bit of land for Lucifer's domain, being determined to cheat the devil after all.

It was in 1825 that James Simpson matriculated at Edinburgh University as a student of arts. At that time his future career was not decided on, but within the two next years he had made up his mind what his future profession was to be, and 1827 found him enrolled among the students of medicine. The expenses of a college education, moderate as they are in Scotch as compared with English Universities, were a serious matter for the lad, even though he was aided by a bursary of £10, tenable for three years. This was not obtained wholly by merit, as by the terms of the deed of gift the trustees were bound to give a preference to candidates called Stewart or Simpson; these being the names of the founders, the Rev. James Stewart, of South Carolina, and his wife. Even with this help, however, strict economy was necessary, and the carefully-kept record of the young student's expenses show that the body's wants, at least, were not luxuriously supplied. "Finnan haddocks, 2d., and bones of the leg, £1 1s.,' stands as the note of one day's expenditure; a proportion which shows us clearly which food, that of body or mind, seemed of the greater importance in his eyes, and gives the key to his nature as clearly as Falstaff's "pennyworth of bread to an intolerable deal of sack" did to his.

While young Simpson was a regular attendant at the classes he took out, and a copious note-taker, he does not seem to have believed all he heard. Frequent marks of interrogation are attached to the report of the professors' utterances, the only notes not thus adorned being those he took in the great surgeon Liston's class. At one time, however, he came near to giving up the profession he had chosen. The sight of pain was horrible to him; and once, after seeing the agony of a poor Highland woman under amputation of the breast, he left the hospital and went to the Signet Office, determined to seek occupation as a lawyer's clerk. On second thoughts, however, he returned to the study of medicine, revolving in his mind the question, "Can anything be done to make operations less painful?"

His mind was directed, early in his studies, to obstetric medicine; he determined that it was the branch of medical science to which he would devote himself. Once, when he escorted some friends to the graduation ceremony, he pointed out to them Dr. Hamilton, the Professor of Midwifery, with words that showed whither his ambition already tended. "Do you see that old man entering? *That's my gown.*"

In April, 1830, before he was nineteen, he took the degree of M.R.C.S.; in 1832 he received the M.D. degree for a thesis on "Death from Inflammation." Before this he had been offered the post of assistant to the Professor of Pathology, at a salary of £50 a year, an income which he made suffice for all wants, even finding means out of it to form the first beginning of his afterwards valuable medical museum. In his student and early graduate days he had been a distinguished member of the Edinburgh Royal Medical Society, an association of students for the purpose of discussing subjects bearing on their future profession. "You are in luck tonight," would be whispered to a visitor when Simpson rose to speak. Already it began to be said that he was a man of genius; but then, and all his life, he protested against the use of that much-abused word. He was fond of quoting a saying of Dr. Armstrong's, that "Genius in a medical man is nothing more than the *habit* of patient observation and reflection."

This is true in a sense; but it may be asked, Whence comes the power of observing and reflecting to any purpose? Many men observe and reflect, and the world is not the richer for it. In medicine, as in all other arts and sciences, to observe the essential, passing over the unimportant, and to reflect justly upon the facts thus noted, is of the very essence of genius. A fool notes many things, often irrelevant, and thinks vaguely about them all; a wise man cares only for those which are permanently true, and using these as premises, reasons from them to a logical conclusion. If one might venture on a new definition of that over-defined yet undefined word *genius*, one would say, Genius is that reflective insight which enables him who possesses it to see the true proportions of any facts he meets with, and the relation they bear to each other. It is this justness of view that marks that "sanity of true genius" which Charles Lamb pointed out, and makes the work of men of genius wholesome and valuable. Vanity may mislead a man of genius into excesses and aberrations; but it is not his genius itself that does so; if it were, then genius would be a hindrance to the progress of humanity—not, as it is, a winged help, which carries a whole generation in a very short time to a point which they would not have reached for many years, or perhaps would never have touched at all.

Helped, then, by genius or hard work, or both, Simpson very soon began to write papers on subjects connected with that branch of his profession which he had chosen, which attracted attention throughout Britain, and on the Continent as well. Several of them were translated into French, German, and Italian. Everything he produced was marked by unusual thoroughness of knowledge and conscientious care. Naturally fond of antiquities, it was a pleasure to him to study the works of old writers as well as new. "In approaching any subject in the literature of his profession," we are told, "his first step was to make himself acquainted with the views and opinions of others; his second, to test these by facts which had come under his own observation." Such a method could not fail to produce good results.

In 1835 Simpson visited the hospitals of London and Paris, returning to Edinburgh with the intention of setting up in practice there, and lecturing on obstetrics in connexion with Dr. Mackintosh, an established extra-mural lecturer. Pecuniary difficulties stood in the way; but the ever-helpful brother Sandy offered to raise the necessary money, and the first course of lectures was delivered with great success in the winter of 1838-39. About this time, also, he turned his attention to mesmerism, and was greatly delighted when he succeeded in his experiments. This was not with him a matter of mere curiosity or delight in the wonderful and mysterious. His mind was still in search of some power that would temporarily and harmlessly deaden sensation, and mesmerism seemed to promise a clue to it. But the anæsthesia of the mesmeric trance was not proof against surgical operations, and when he perceived this, Simpson let the subject drop.

In 1839 came a turning-point in his life. Dr. Hamilton resigned the chair of Midwifery, and Simpson became a candidate for it. There was a strong opposition to his election; every possible defect was proved against him. Some said he was too young, some complained of his being unmarried, some even showed such a petty spirit as to protest against "the baker's son" being raised to the dignity of a professorship in the University of Edinburgh. His opponents all agreed that for some reason or another he was not the man for the post; the only thing they dared not say was that he was inferior to the other candidates in intellect, energy, or conscientious devotion to his work. The opposition, and especially the acrimonious and personal spirit in which it was carried on, roused all the pugnacity of James Simpson's nature—an ample supply! Too young! Youth was a fault that mended soon enough, and it was less disqualifying to be too young for a post than too old for it. Unmarried! That could easily be remedied. He had already chosen his wife, Jessie Grindlay, a cousin of his own, though the insecurity of his prospects had hitherto prevented his speaking of his hopes; but compulsion to such a step was not unwelcome. And the baker's son proved himself in the contest somewhat more of a gentleman than many of his well-born antagonists. He fought for the post he desired, it is true, and fought bravely, using every influence he could avail himself of; but he took no underhand means, and did not indulge in unworthy disparagement of the other candidates, though he stoutly maintained his conviction that the work he had already done in his profession made him as fit a man as any of them for the professorship.

